# p62 acts as an oncogene and is targeted by miR-124-3p in glioma

**DOI:** 10.1186/s12935-019-1004-x

**Published:** 2019-11-06

**Authors:** Danni Deng, Kaiming Luo, Hongmei Liu, Xichen Nie, Lian Xue, Rong Wang, Yuan Xu, Jun Cui, Naiyuan Shao, Feng Zhi

**Affiliations:** 1 Department of Neurosurgery, The First People’s Hospital of Changzhou, #185 Juqian Road, Changzhou, Jiangsu China; 2 Modern Medical Research Center, The Third Affiliated Hospital of Soochow University, #185 Juqian Road, Changzhou, Jiangsu China; 3 Department of Endocrinology, The First People’s Hospital of Changzhou, Changzhou, Jiangsu China; 4 School of Biological Science and Medical Engineering, Beihang University, #37 Xueyuan Road, Beijing, China; 5 MOE Key Laboratory of Gene Function and Regulation, State Key Laboratory of Biocontrol, School of Life Sciences, Sun Yat-sen University, #135 Xingangxi Road, Guangzhou, China

**Keywords:** p62, miR-124-3p, Proliferation, TMZ resistance, Glycolysis, Migration

## Abstract

**Background:**

Glioma is the most common central nervous system (CNS) tumour. p62, an important autophagy adaptor, plays a crucial role in cancer. However, the role of p62 in the progression of glioma is poorly characterized.

**Methods:**

We examined the expression of p62 in glioma tissues and cell lines. Then we investigated the function of p62 in vitro, and clarified the mechanism underlying the regulation of p62 expression.

**Results:**

We revealed that p62 was upregulated at both the mRNA and protein levels in human glioma tissues irrelevant to isocitrate dehydrogenase (IDH) status. Then, we found that overexpression of p62 promoted glioma progression by promoting proliferation, migration, glycolysis, temozolomide (TMZ) resistance and nuclear factor κB (NF-κB) signalling pathway, and repressing autophagic flux and reactive oxygen species (ROS) in vitro. In accordance with p62 overexpression, knockdown of p62 exerted anti-tumour effects in glioma cells. Subsequently, we demonstrated that miR-124-3p directly targeted the 3′-UTR of p62 mRNA, leading to the downregulation of p62. Finally, we found that p62 function could be partially reversed by miR-124-3p overexpression.

**Conclusions:**

Our results demonstrate that p62 can be targeted by miR-124-3p and acts as an oncogene in glioma, suggesting the potential value of p62 as a novel therapeutic target for glioma.

## Background

As one of the most common and lethal central nervous system (CNS) tumours, gliomas are classified into four grades (I–IV) by the World Health Organization based on histopathology and molecular pathways [1]. Unfortunately, malignant gliomas (grade III to IV), which are associated with a dismal prognosis, constitute the majority of gliomas. Currently, the standard treatment strategy for malignant glioma is maximal safe surgical resection followed by radiotherapy and/or adjuvant chemotherapy with temozolomide (TMZ). Despite recent clinical advances, the median survival time of patients diagnosed with malignant glioma is 1–2 years [2]. Hence, a deep understanding of the molecular mechanisms driving tumorigenesis and progression may provide candidates for more effective therapies for the treatment of malignant glioma.

p62, encoded by SQSTM1, is the most well-known autophagy adaptor, and it plays a crucial role in both normal physiology and cancer. Accumulating evidence demonstrates that aberrant p62 expression is associated with aggressive clinicopathologic features and poor prognosis in cases of pancreatic cancer, oral squamous cell carcinoma, metastatic breast cancer and hepatocellular carcinomas (HCC) [3–5]. p62 accumulation, induced by inflammation or autophagy impairment, promotes the initiation and progression of cancer through repressing apoptotic resistance and reactive oxygen species (ROS) generation and enhancing cell proliferation, survival, tumorigenesis and metastasis [4–6]. Additionally, high levels of p62 promote radiotherapy and chemotherapy resistance in hypopharyngeal carcinomas and ovarian cancer [7, 8]. Research on p62 aberrance in the CNS has mainly focused on neurodegenerative disorders, such as Parkinson’s disease and Alzheimer’s disease, while the role of p62 in the progression of glioma is poorly characterized.

MicroRNAs (miRNAs) are endogenous, single-stranded, non-coding RNAs of approximately 19–25 nucleotides, and approximately 30% of protein-coding genes might be regulated by miRNAs [9]. MiRNAs can directly bind to complementary sequences in the mRNA 3′-UTR, resulting in translation inhibition or mRNA degradation [10]. MiR-124-3p, a brain-enriched miRNA, is either minimally expressed or absent and acts as a tumour suppressor in glioma, suggesting that miR-124-3p may be a novel diagnostic biomarker and therapeutic target in glioma [9]. Furthermore, miR-124-3p can inhibit glioma progression through repressing proliferation, invasion and the stem-like traits, and potentiating chemosensitivity by targeting different genes [11, 12]. In our previous work, we identified the anti-tumour effect of miR-124-3p in the pathogenesis of astrocytoma by targeting PIM1 [13].

Our study reveals the expression pattern and functions of p62 in glioma and further clarifies the regulatory mechanism between miR-124-3p and p62, suggesting the potential value of p62 as a therapeutic target for the treatment of glioma.

## Materials and methods

### Human tissues

In total, 26 (normal tissue = 10, grade II = 4, grade III = 6, grade IV = 6) tissue samples were collected from the Department of Neurosurgery at the Third Affiliated Hospital of Soochow University (Changzhou, China). Each patient signed an informed consent, and the study was approved by the Research Ethics Board of the Third Affiliated Hospital of Soochow University. Ten normal tissue samples were obtained from patients with cranial trauma resulting from traffic accidents. The patient cohort consisted of 9 males and 7 females. Of these patients, 10 were older than 50 years of age, while 6 were younger. Histological diagnoses were confirmed by three independent pathologists according to the WHO classification [1]. The tissues were immediately snap-frozen and stored in liquid nitrogen for further analysis.

### Cell lines and cell culture

HEK293T, the human glial cell HEB and three glioma cell lines, including U87, LN229 and U251 were obtained from the Type Culture Collection of the Chinese Academy of Sciences (Shanghai, China). Cells were cultured in Dulbecco’s Modified Eagle’s medium (Gibco, USA) with 10% fetal bovine serum (FBS, Gibco, USA) at 37 °C with 5% CO_2_.

### Cell transfection

Synthetic miR-124-3p mimic (pre-miR-124-3p), mimic negative control (pre-ncRNA), miR-124-3p inhibitor (anti-miR-124-3p) and inhibitor negative control (anti-ncRNA) were purchased from GenePharma (Shanghai, China).

Synthetic p62 siRNAs and negative control siRNA (nc-siRNA) were purchased from GenePharma (Shanghai, China). The sequences of these oligonucleotides are shown in Additional file 1: Table S1. The vector containing the full-length open reading frame (ORF) of p62 without its 3′-UTR and the negative control vector (nc-vector) were purchased from Genewiz (Soochow, China).

All transient transfections were carried out with Lipofectamine™ 2000 Transfection Reagent (Invitrogen, USA) according to the manufacturer’s specifications.

### Immunohistochemistry staining

Immunohistochemical (IHC) analyses were performed to study p62 protein expression in the 26 tissues. The tissues were embedded in paraffin and then cut into 6-µm-thick sections. These sections were dewaxed in xylene and then rehydrated in an ethanol series. Pressure cooking in citrate buffer (pH = 6) (Beyotime, China) for 5 min was applied to for antigen retrieval. Next, blocking of endogenous peroxidase was performed in 0.3% H_2_O_2_ for 10 min. Incubation with the p62 primary antibody (dilution 1:1000, AB56416, Abcam, USA) occurred overnight at 4 °C. Detection was performed with HRP-labelled goat anti-mouse IgG (dilution 1:2000, A0216, Beyotime, China) for 30 min, followed by incubation with the DAB chromogen with a DAB horseradish peroxidase chromogen kit (Beyotime, China). Sections were counterstained in haematoxylin (Beyotime, China) for 10 s and dehydrated in an ethanol series.

### Mutation analysis of isocitrate dehydrogenase (IDH)

Genomic DNA was extracted from frozen tissues using DNA extraction Kit (Tiangen, China), followed by quality evaluation on Nanodrop 1000 Spectrophotometer (Thermo Fisher, USA). The genomic regions which encompassed codons R132 of IDH1 and R172 of IDH2 were amplified by PCR and the sequences of primers are shown in Additional file 2: Table S2. Sanger sequencing reactions were performed by Genewiz, Inc. (Soochow, China).

### Fluorescence in situ hybridization analysis of 1p/19q codeletion

Four oligodendroglioma tissues were embedded in paraffin and then cut into 6-µm-thick sections 1p/19q codeletion was detected with 1p36/1q25 and 19q13/19p13 Dual-Color Probe kit (Abbott Molecular Inc, USA) according to the manufacturer’s specifications.

### RNA isolation and quantitative real-time PCR

Total RNA was extracted from frozen tissues using TRIzol Reagent (Invitrogen, USA) according to the manufacturer’s specifications. TaqMan miRNA probes (Thermo Fisher Scientific, USA) were used to quantify the level of miR-124-3p on the ABI 7500 System (Thermo Fisher Scientific, USA), as previously reported [13]. We used quantitative real-time PCR (qRT-PCR) to quantify the mRNA level of p62, C–C motif chemokine ligand 2 (CCL2), transforming growth factor beta 1 (TGFβ1), colony stimulating factor 3 (CSF3) and interleukin 6 (IL-6) with SYBR Green PCR Master Mix (Takara, Japan) according to the manufacturer’s instructions. U6 and β-actin served as the endogenous controls to miRNA and mRNA. The probes were purchased from Applied Biosystems (Thermo Fisher Scientific, USA), and the primers were purchased from Genewiz (Soochow, China). The sequences are shown in Additional file 2: Table S2.

### Western blot analysis

Total protein was extracted from frozen tissues and western blot was carried out as previously described [13]. The following antibodies were used: p62 (dilution 1:1500, 5114), β-actin (dilution 1:3000, 3700), Lamin B1 (dilution 1:1000, 13,435), nuclear factor κB (NF-κB) p65 (dilution 1:1000, 8242), HRP-labelled goat anti-mouse IgG (dilution 1:3000, A021) and HRP-labelled goat anti-rabbit IgG (dilution 1:3000, A0208). The yields of LC3 I and II was detected with LC3B antibody (dilution 1:2000, 3868). All the primary antibodies were purchased from Cell Signaling Technology (USA), and all the secondary antibodies were purchased from Beyotime (China). Protein bands were visualized with the ECL Advanced Western Blot Detection Kit (Thermo Fisher Scientific, USA) on the Bio-Rad ChemiDoc™ Touch (Bio-Rad, USA) and quantified with Image Lab (Bio-Rad, USA) software.

### Luciferase assay

The wild-type 3′-UTR of the p62 luciferase reporter vector was constructed by amplifying the miR-124-3p binding sites in the p62 mRNA 3′-UTR and inserting them into the pMIR-REPORT™ plasmid (Thermo Fisher Scientific, USA). A mutant vector was constructed to test for binding specificity. Next, 20 μg reporter vectors and 10 μg control Renilla luciferase plasmid pRL-SV40 (Promega, USA) were co-transfected into HEK293T cells 24 h before testing. The luciferase assay was performed using the Dual-Luciferase^®^ Reporter Assay System (Promega, USA) according to the manufacturer’s specifications.

### Wound healing assay

The wound healing assay was carried out as previously reported [13]. At various time points, the plates were photographed with an IX71 microscope (Olympus, USA), and the distance was measured by ImageJ.

### Cell proliferation assay

Transfected cells were seeded at 3000 cells per well in 96-well plates. Cell proliferation was assayed using Cell-Counting Kit 8 (CCK-8, Beyotime, China) at 0, 24, 48 and 72 h, according to the manufacturer’s specifications. The absorbance at a wavelength of 450 nm was measured with the BioTek Elx800 (BioTek, USA).

Relative TMZ toxicity was evaluated with a cell proliferation assay; 24 h after seeding, the cells were then treated with 400 µM TMZ (Sigma, USA) or an equivalent solvent for 48 h. After incubation with TMZ, the CCK-8 (Beyotime, China) assay was applied to investigate the relative TMZ toxicity.

### Migration assay

As previously reported, transwell plates were used to assess cell migration in vitro [13]. The chambers were then photographed using an IX71 microscope (Olympus, USA) in six randomly selected fields. The number of migrated cells in every picture was counted.

### ROS assay

Cellular ROS was assayed using a reactive oxygen species assay kit (Beyotime, China) according to the manufacturer’s specifications and examined on the Guava EasyCyte 6HT-2L flow cytometer (Millipore, USA).

### Apoptosis assay

Cells were incubated in 400 mM TMZ or an equivalent solvent for 48 h. After incubation with TMZ, apoptosis was assayed with an Annexin V-FITC apoptosis detection kit (Beyotime, China) to investigate the effects of TMZ on cell apoptosis on the Guava EasyCyte 6HT-2L flow cytometer (Millipore, USA) according to the manufacturer’s specifications.

### Glycolysis assay

The extracellular acidification rate (ECAR) was measured using a Seahorse XF-96 extracellular flux analyzer (Agilent, USA), and the experiment was carried out as previously described [13]. The ECAR was automatically calculated by Wave (Agilent, USA). Each data point represented the average of eight wells.

### Statistics

All experiments were independently performed at least 3 times, and the means and standard error or standard deviation were subjected to the Student’s t test for pair wise comparison or ANOVA for multivariate analysis using GraphPad Prism software.

## Results

### p62 is overexpressed in glioma

**Fig. 1:**
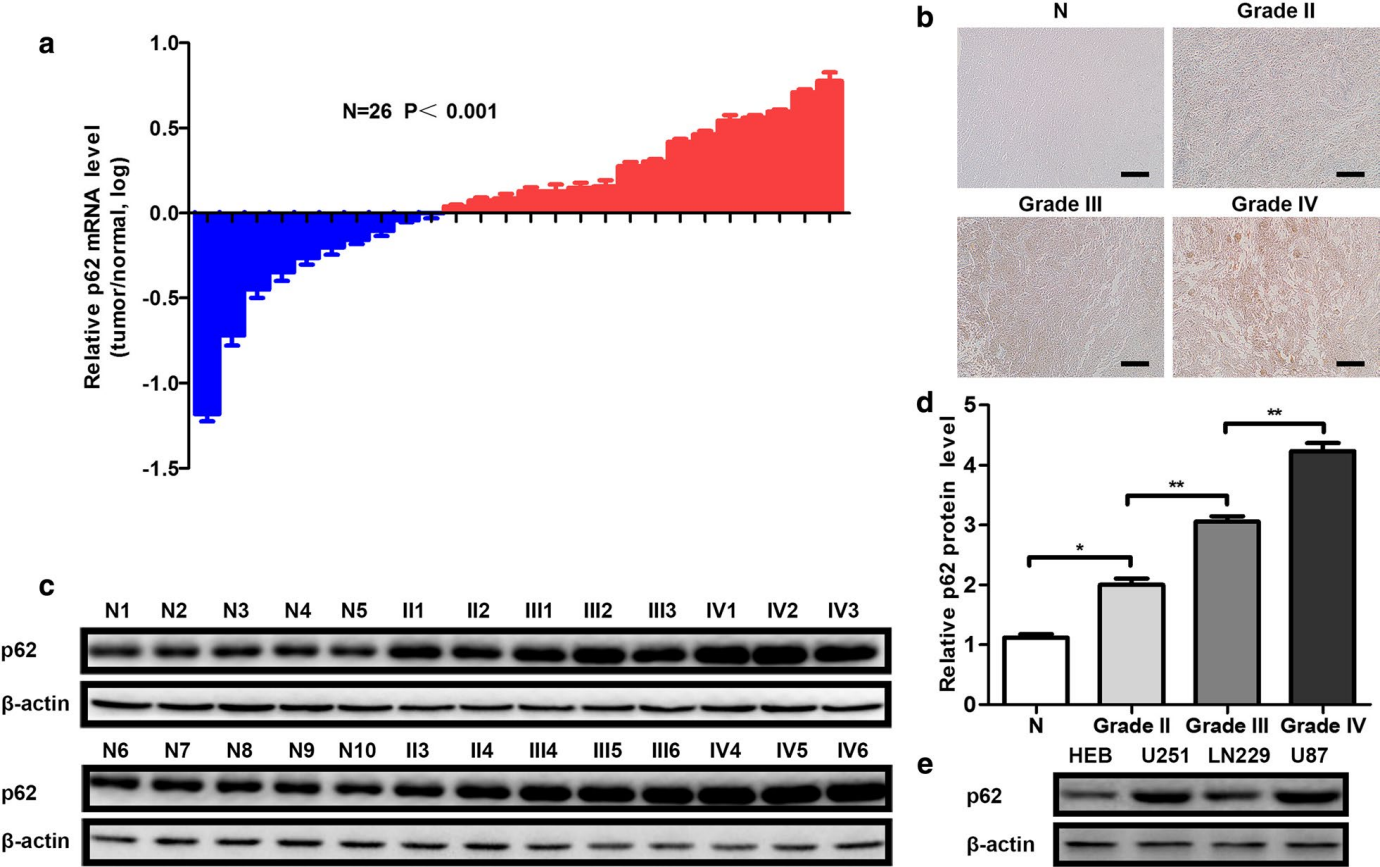
p62 is overexpressed in glioma. **a** Relative mRNA levels of p62 in 26 tissues. The blue shading indicates normal brain tissues, and the red shading indicates glioma tissues. **b** Representative results of p62 IHC in tissues with different malignance. N indicates normal brain tissues. Each scale bar indicates 400 μm. **c** Protein levels of p62 in 26 tissues detected with western blot. T indicates glioma tissues. **d** Statistical analysis for protein levels of p62 in 26 tissues detected with western blot. **e** Protein levels of p62 in different cell lines detected with western blot. *P < 0.05, **P < 0.01

To investigate the relationship between glioma malignancy and the expression of p62, we analysed the expression of p62 with IHC, western blot and qRT-PCR. First, we used qRT-PCR to qualify the expression of p62 mRNA in 10 normal tissues and 16 glioma tissues. 9 glioma tissues were IDH mutant and 7 were IDH wild-type. Detailed clinical and molecular characteristics of glioma patients were shown in Table 1. The relative mRNA levels of p62 were obviously upregulated in gliomas (Fig. 1a). Subsequently, we confirmed that accumulated p62 proteins were detected in the glioma samples, and p62 expression increased progressively from WHO grade II to IV (Fig. 1b–d). At last, we investigated the relative protein levels of p62 in human glial cell HEB and three glioma cell lines, including U87, LN229 and U251, and found p62 was overexpressed in glioma cell lines, compared with HEB (Fig. 1e). Furthermore, we found that expression of p62 had no correlation with IDH mutation status, indicating that the effect of p62 might not depend on IDH status (Additional file 3: Figure S1A, B). We decided to choose U87 and U251 glioma cell lines for further study. Our data suggest that p62 is closely involved in glioma progression and may be an oncogene in gliomas.

**Table 1:** Clinical and molecular characteristics of glioma patients

Parameters	WHO grade II	WHO grade III	WHO grade IV
Number of patients	4	6	6
Gender			
Male	2	3	3
Female	2	3	3
Mean age (years)	48.6 ± 4.9	49.3 ± 3.8	52.9 ± 5.5
Histopathology			
Astrocytoma	3	3	0
Oligodendroglioma	1	3^a^	0
Glioblastoma	0	0	6
IDH status			
Mutated	3	4	2
Wild type	1	2	4

^a^Two of the three oligodendroglioma tissues were 1p/19q co-deleted

### p62 overexpression promotes cancer progression in glioma cells

**Fig. 2:**
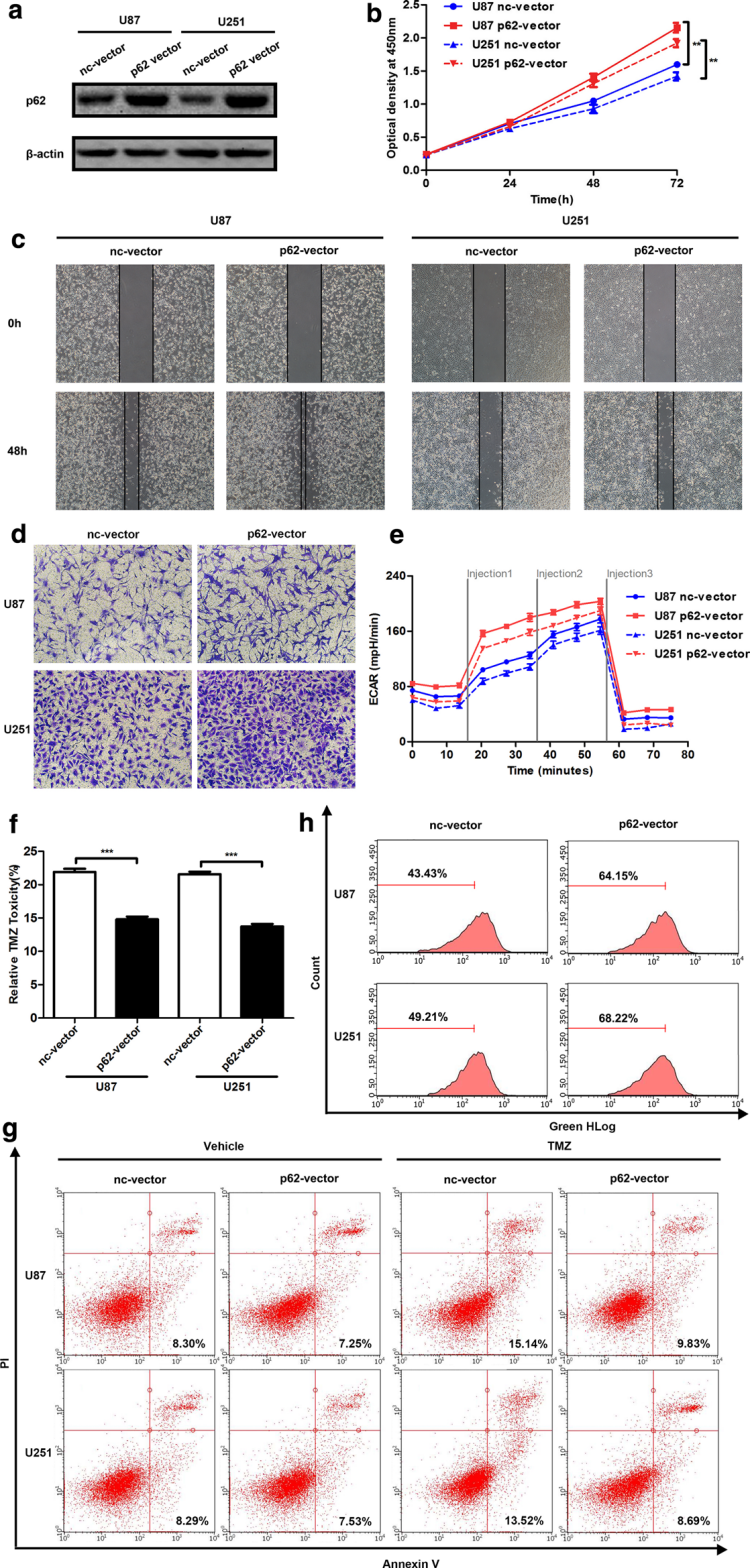
p62 overexpression promotes cancer progression in glioma cells. **a** p62 protein levels in U87 and U251 cells after transfection with nc-vector or p62-vector detected with western blot. **b** Role of p62 overexpression in cell proliferation. **c** Representative results of wound healing assays in p62-overexpressed cells (×100 magnification). **d** Representative results of the transwell assay in p62-overexpressed cells (200 × magnification). **e** Role of p62 overexpression in glycolysis. **f** Role of p62 overexpression in TMZ resistance detected with CCK8 assay. **g** Representative results of apoptosis induced by TMZ in p62-overexpressed cells. **h** Representative results of ROS in p62-overexpressed cells. **P < 0.01, ***P < 0.001

For the investigation of the function of aberrant p62 expression in glioma cells, p62 was overexpressed (Fig. 2a) by transiently transfecting U87 and U251 cells with the p62-overexpressing plasmid (p62-vector). The results of the cell proliferation assay indicated that p62 overexpression significantly promoted cell growth in U87 and U251 glioma cells (Fig. 2b). Since p62 is an important adaptor in autophagy, we treated transfected cells with chloroquine (CQ), an autophagy inhibitor, to evaluate the effect of p62 on autophagic flux. After incubation with 10 μM CQ for 24 h, total protein was extracted for a western blot assay. The LC3 II/LC3 I ratio in the CQ-treated p62-vector group was slightly lower than the CQ-treated negative control vector (nc-vector) group in U87 cells, indicating that p62 overexpression could inhibit autophagic flux in U87 (Additional file 4: Figure S2A and B). Next, the wound healing assay was conducted to investigate the cell migration and invasion capabilities. The results showed that accumulation of p62 significantly increased the speed of wound closure in U87 and U251 cells (Fig. 2c). We then adapted a transwell assay to evaluate the role of p62 overexpression in cell migration. The overexpression of p62 markedly enhanced the migration capacity of U87 and U251 cells (Fig. 2d). Then we measured ECAR, an indicator of lactic acid production through glycolysis, to evaluate the effect of p62 overexpression on cellular energetics. A substantial promotion was observed in the p62-vector group, as shown by a significant increase in ECAR compared with that in the nc-vector group (Fig. 2e). Furthermore, the influence of p62 accumulation in TMZ resistance was investigated by CCK-8 and cell apoptosis assays. Relative TMZ toxicity was significantly decreased in the p62-vector group compared with that in the nc-vector group (Fig. 2f). In accordance with the CCK-8 assay results, p62 overexpression could protect U87 and U251 cells from apoptosis induced by TMZ (Fig. 2g). Then we evaluated the effect of p62 overexpression on oxidative stress by measuring the reactive oxygen species in transfected cells, and the results showed that p62 accumulation caused mild decreases in the levels of reactive oxygen species (Fig. 2h). We also investigated the effects of p62 overexpression in NF-κB signalling pathway. The level of nuclear NF-κB was slightly increased in p62 overexpression group, compared with nc-vector group (Additional file 4: Figure S2C). Several NF-κB downstream target genes including CCL2, IL-6, TGFβ1 and CSF3 were examined. As shown in Additional file 4: Figure S2D–G, these genes were activated after p62 overexpression. Taken together, our data demonstrate that accumulated p62 can regulate cell proliferation, autophagy, wound healing, cell migration, glycolysis, TMZ resistance, oxidative stress and activate NF-κB signalling pathway, suggesting its oncogene role in gliomas.

### p62 knockdown represses cancer progression in glioma cells

**Fig. 3:**
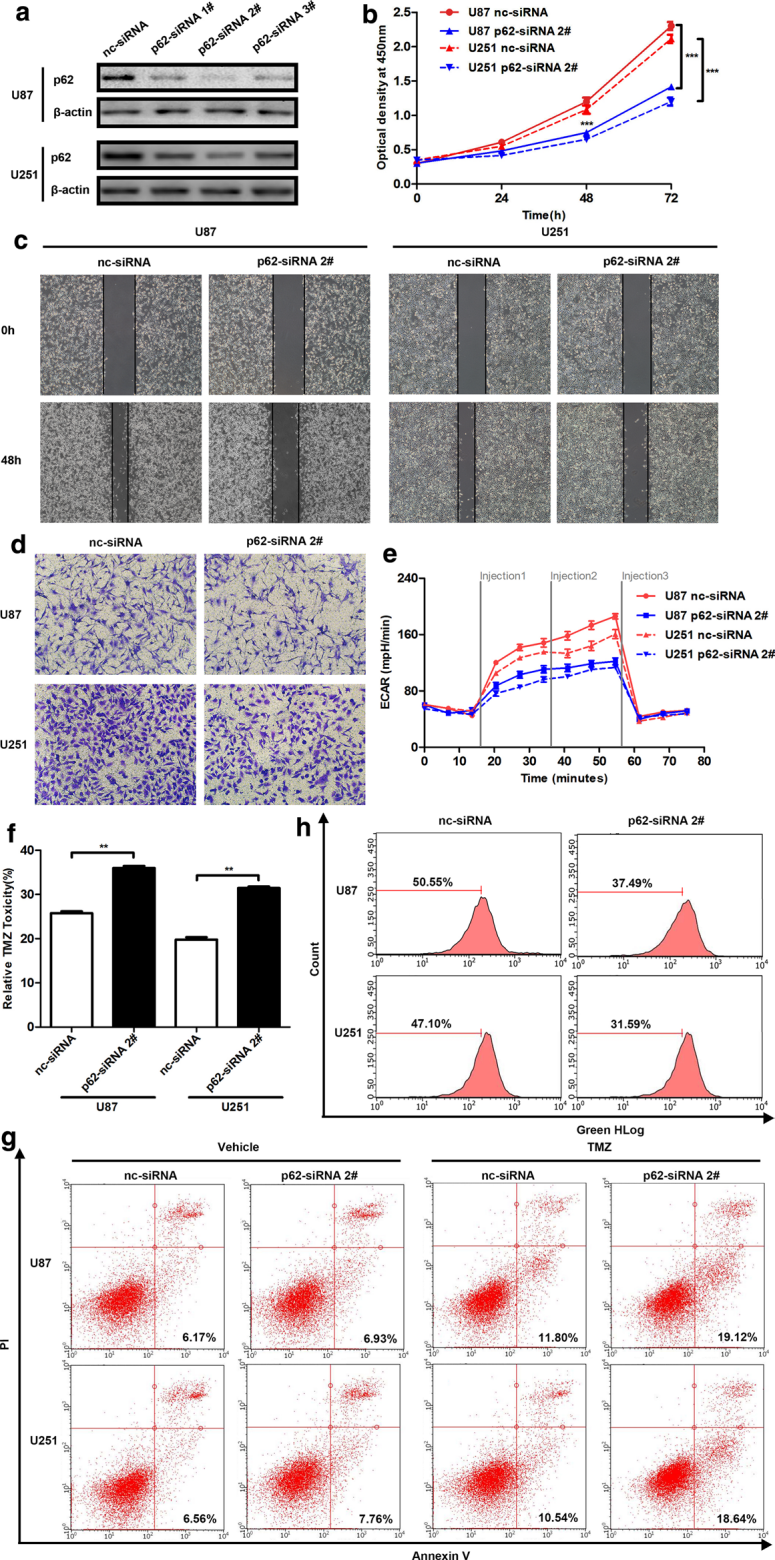
p62 knockdown represses cancer progression in glioma cells. **a** Knockdown efficiency of p62 siRNAs in U87 and U251 cells detected with western blot. **b** Role of p62 knockdown in cell proliferation. **c** Representative results of wound healing assays in p62 knockdown cells (×100 magnification). **d** Representative results of the transwell assay in p62 knockdown cells (×200 magnification). **e** Role of p62 knockdown in glycolysis. **f** Role of p62 knockdown in TMZ resistance detected with CCK8 assay. **g** Representative results of apoptosis induced by TMZ in p62 KD cells. **h** Representative results of ROS in p62 knockdown cells. **P < 0.01, ***P < 0.001

Given that p62 overexpression could promote glioma, we generated p62 knockdown (KD) cells with siRNAs to investigate whether downregulation of p62 could repress glioma cells. The western blot results showed that the siRNAs could knockdown the expression of p62 protein efficiently, especially p62 siRNA 2# (Fig. 3a). CCK-8 results indicated that p62 knockdown significantly repressed cell growth (Fig. 3b). Next, the influence of p62 knockdown on autophagic flux was tested. There was no significant difference in the LC3 II/LC3 I ratio between the CQ-treated p62 KD group and the CQ-treated nc-siRNA group, indicating that p62 knockdown had no effect on autophagic flux in glioma cells (Additional file 5: Figure S3A, B). Subsequently, the migration and invasion capabilities were assessed by wound healing and transwell assay, and the results indicated that wound closure speed (Fig. 3c) and migration capacity (Fig. 3d) were significantly decreased in the p62 KD groups. Glycolysis was also measured, and there was a substantial block in glycolysis due to p62 knockdown (Fig. 3e). Next, TMZ resistance in p62 KD cells was evaluated. p62 KD cells were more sensitive to TMZ treatment (Fig. 3f, g). Finally, the reactive oxygen species were tested in p62 KD cells, and the results showed that p62 knockdown enhanced the levels of reactive oxygen species (Fig. 3h). Finally, as expected p62 knockdown inhibited nuclear NF-κB expression and its downstream target genes (Additional file 5: Figure S3D–G). In sum, the loss of p62 functions opposite to p62 accumulation, thereby exerting anti-tumour effects in glioma.

### p62 is directly targeted by miR-124-3p in glioma

**Fig. 4:**
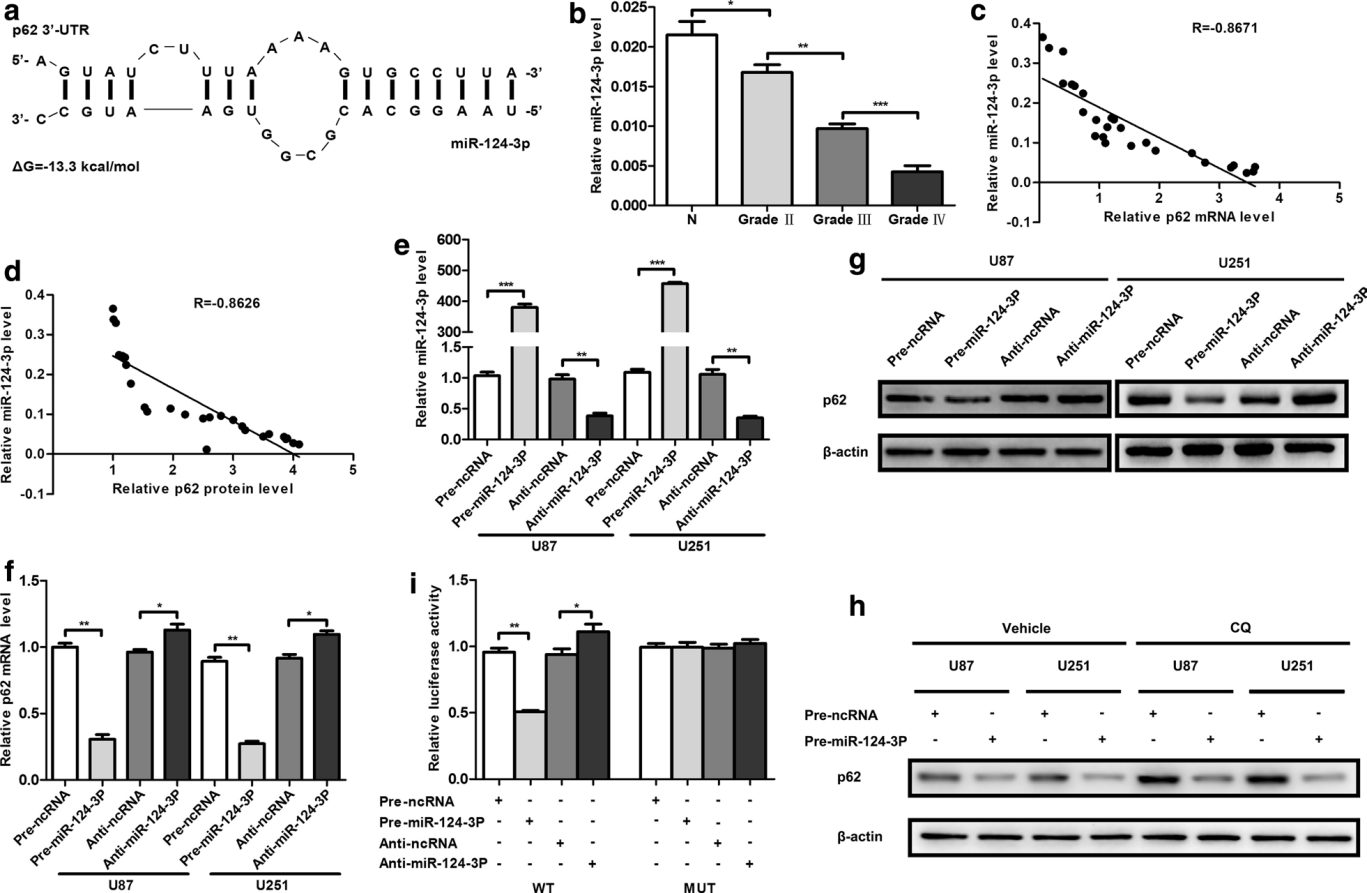
p62 is directly targeted by miR-124-3p in glioma. **a** Schematic illustration of the predicted interactions between the p62 3′-UTR and miR-124-3p with free energy indicated. **b** Relative levels of miR-124-3p in 26 tissues. **c** Pearson’s correlation for relative p62 mRNA levels and relative miR-124-3p levels in 26 samples. **d** Pearson’s correlation for relative p62 protein levels and relative miR-124-3p levels in 26 samples. **e** Relative levels of miR-124-3p after transfection with pre-miR-124-3p, anti-miR-124-3p or control oligonucleotides. **f** Relative mRNA levels of p62 after transfection with pre-miR-124-3p, anti-miR-124-3p or control oligonucleotides. **g** Relative protein levels of p62 after transfection with pre-miR-124-3p, anti-miR-124-3p or control oligonucleotides. **h** Relative protein levels of p62 after transfection with pre-miR-124-3p or control oligonucleotides and treatment with CQ. **i** Luciferase assay for miR-124-3p and p62 3′-UTR. *P < 0.05, **P < 0.01, ***P < 0.001

We used TargetScan, a computational algorithm, to search for potential miRNAs targeting p62. The seed sequence of miR-124-3p matched 3′-UTR of p62, and the minimum free energy of the hybrids was − 13.3 kcal/mol (Fig. 4a). As previously reported, the expression of miR-124-3p was significantly downregulated in glioma tissues, decreasing progressively from WHO grade II to IV (Fig. 4b) [13]. Furthermore, the relative levels of both p62 mRNA and protein increased as the relative miR-124-3p level decreased, suggesting that miR-124-3p might regulate p62 expression through both post-transcriptional mechanisms and mRNA stability (Fig. 4c, d).

For further investigation of the effect of miR-124-3p on p62 expression, miR-124-3p mimic and inhibitor were transfected into U87 and U251 cells. The expression of miR-124-3p significantly increased after transfection with pre-miR-124-3p and moderately decreased after transfection with anti-miR-124-3p (Fig. 4e). To verify whether miR-124-3p affected p62 expression at both the mRNA and protein levels, we extracted the total RNA and protein contents of U87 and U251 cells and examined them with qRT-PCR and western blot, respectively, after transfection with pre-miR-124-3p and anti-miR-124-3p. MiR-124-3p overexpression significantly reduced the relative p62 mRNA level by approximately 65%, and miR-124-3p inhibition increased the relative p62 mRNA level by approximately 15% (Fig. 4f). The reduction in p62 protein level induced by miR-124-3p overexpression was approximately 45% (Fig. 4g). Since p62 was an important adaptor in autophagy and could be degraded along with autophagy, CQ was used to eliminate the potential influence of miR-124-3p on autophagic flux. The relative p62 protein level decreased with increases in the miR-124-3p dose, even in the CQ treatment group, indicating that miR-124-3p overexpression could repress the expression of p62 directly (Fig. 4h).

A luciferase assay was applied to investigate whether the influence of miR-124-3p on the expression levels of p62 was mediated through the predicted binding between miR-124-3p and mRNA 3′-UTR of p62. The results showed that pre-miR-124-3p could repress the luciferase activity compared with pre-ncRNA, while anti-miR-124-3p enhanced luciferase activity (Fig. 4i). The mutant firefly luciferase reporter plasmids, however, had no effect on luciferase activity in either the pre-miR-124-3p or pre-ncRNA groups. These results indicate that the p62 mRNA 3′-UTR is directly targeted by miR-124-3p, leading to the downregulation of p62 expression at both the mRNA and protein levels.

### p62 function is partially reversed by miR-124-3p

**Fig. 5:**
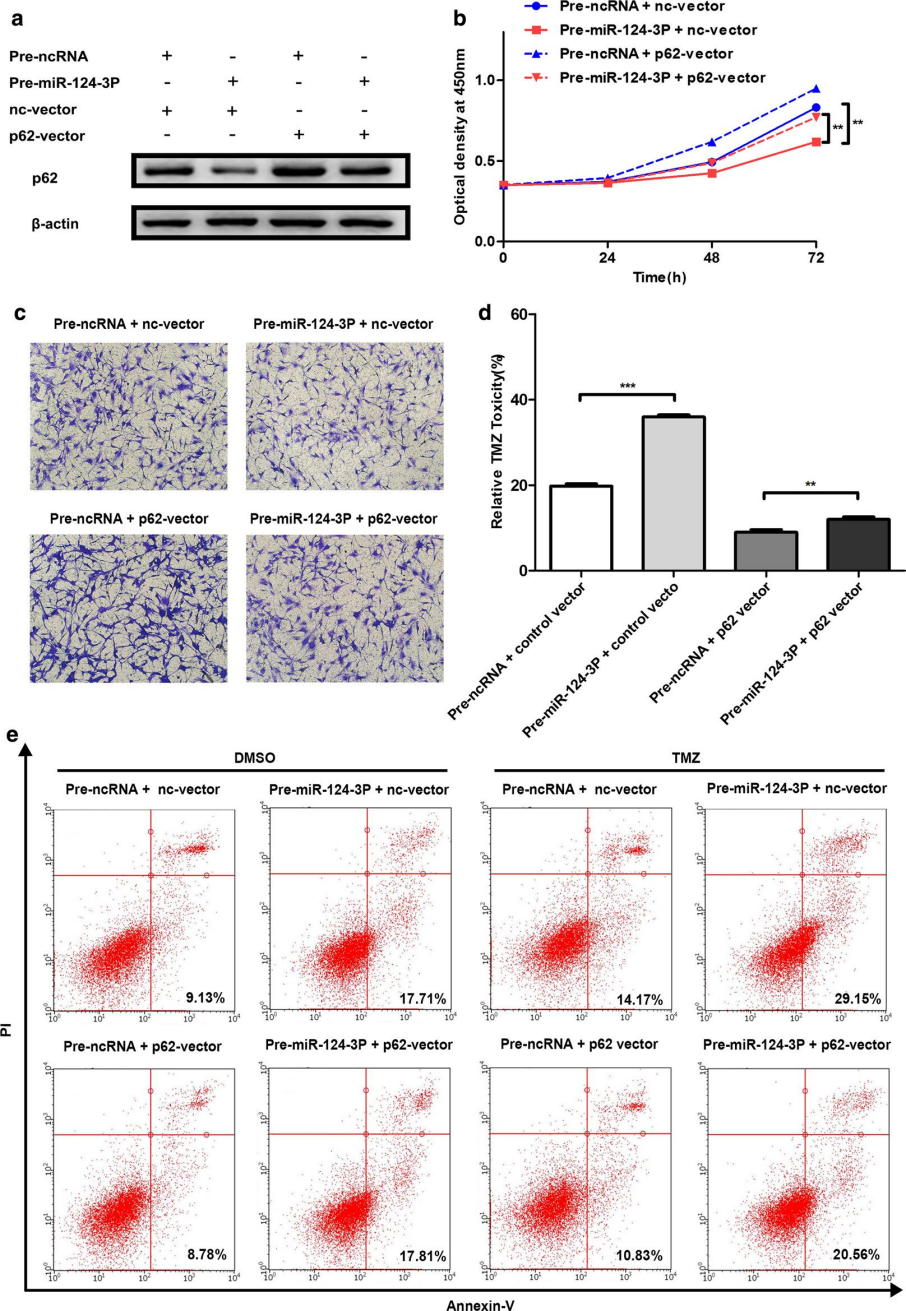
p62 function is partially reversed by miR-124-3p. **a** Relative protein levels of p62 in U87 cells transfected with nc-vector or p62-vector, along with pre-ncRNA or pre-miR-124-3p. **b** Role of ectopic p62 and miR-124-3p expression in cell proliferation. **c** Role of ectopic p62 and miR-124-3p expression in cell migration (×200 magnification). **d** Role of ectopic p62 and miR-124-3p expression in TMZ resistance detected with CCK8 assay. **e** Representative results of apoptosis induced by TMZ in p62- and miR-124-3p-overexpressed cells. **P < 0.01, *** *P *< 0.001

Since the function of p62 and miR-124-3p was similar in U87 and U251, we investigated the influence of miR-124-3p on p62 function in U87 glioma cell line only. Firstly, we used a western blot test to detect the integrative effect of p62 and miR-124-3p overexpression on the p62 protein level. p62 accumulation could be partially reversed by miR-124-3p overexpression (Fig. 5a). Then, we performed the CCK-8 assay, the cell migration assay and the TMZ resistance assay to examine whether the introduction of miR-124-3p could reverse the functions of p62. The ectopic expression of miR-124-3p partially reversed the promotion of proliferation (Fig. 5b), migration (Fig. 5c) and TMZ resistance (Fig. 5d, e) induced by p62 overexpression, indicating that miR-124-3p exerted anti-tumour effects by suppressing p62 expression through binding to the p62 mRNA 3′-UTR directly. We revealed the function of p62 in glioma cells and further examined the interaction between p62 and miR-124-3p to clarify the underlying mechanism of p62 in glioma.

## Discussion

In this paper, p62 was identified as aberrantly upregulated at both the mRNA and protein levels in human glioma tissues irrelevant to IDH status. We then focused on the functions of p62 and demonstrated that the accumulation of p62 could promote glioma by regulating autophagy, proliferation, migration, reactive oxygen species, TMZ resistance, glycolysis and NF-κB signalling pathway. In accordance with p62 overexpression, p62 knockdown exerted anti-tumour effects in U87 and U251 glioma cells. Subsequently, we determined that miR-124-3p directly targeted the mRNA 3′-UTR of p62, leading to the downregulation of p62 expression at both the mRNA and protein levels. Furthermore, p62 function could be partially reversed by miR-124-3p overexpression. Our findings suggest the potential value of p62 as a novel therapeutic target for glioma.

p62 is a multi-domain protein and thus exerts diverse functions through interacting with different molecules. First, p62 binds to mitogen-activated protein kinase kinase kinase 3 (MEKK3) through an N-terminal oligomerization domain (PB1), which leads to mTORC1 activation and c-Myc expression, thus promoting cancer cell proliferation in prostate cancer stromal fibroblasts and HCC [5, 14]. In addition, p62 binds to LC3 on phagophore membranes through the LC3-interacting region (LIR) and delivers ubiquitylated cargos to the autophagosome for further degradation [15]. GATA4, a tumour suppressor, is degraded by p62-dependent selective autophagy and is confirmed to be downregulated in GBM [16]. Re-expression of GATA4 mediates sensitization of GBM cells to TMZ treatment through loss of APNG (alkylpurine-DNA-*N*-glycosylase), a poorly characterized DNA repair enzyme, instead of O-6-methylguanine-DNA-methyltransferase (MGMT) [17]. Moreover, p62 binds TNFR-associated factor 6 (TRAF6) with the TRAF6-binding domain (TB) to activate NF-κB signalling, resulting in the expression of inflammatory genes and cancer metastasis [8, 18]. (4) Additionally, with the Keap1-interacting region (KIR), p62 binds to Kelch-like ECH-associated protein 1 (Keap1), leading to the activation of nuclear factor E2 related factor 2 (Nrf2), a transcription factor responsible for several antioxidant genes, which subsequently maintains a low level of ROS to protect cancer cells from oxidative damage [5, 6, 19]. Constitutive p62 and Nrf2 overexpression is detected in many tumour types and can favour cancer cell survival, promote cell proliferation and protect tumour cells from chemotherapy, radiotherapy and oxidative stress [6]. The overexpression of Nrf2 and p62 is observed in glioma samples and is identified to be closely related with the clinicopathological parameters and prognosis of patients with gliomas [20]. Finally, p62 knockdown cells exhibited decreased lactate secretion and glucose uptake through the mTORC1/c-Myc pathway and F1F0-ATP synthase dysfunction [14, 21]. On the other hand, high p62 expression induces high hexokinase 2 and hypoxia-inducible factor α expression via the upregulation of mTORC1 and NF-κB activity and the inhibition of von Hippel–Lindau E3 ubiquitin ligase activity, leading to enhancement of glycolysis in cancer [22, 23]. In our research, we have found that p62 acts as a tumour promotor by regulating autophagy, proliferation, migration, reactive oxygen species, TMZ resistance-, glycolysis and NF-κB signalling pathway in glioma cells. However, it has to be noted that the control for p62-overexpressing plasmid is an empty vector in our experiment which may cause the cells grow faster than other control, since DNA length can also affect the cell growth. This may be the reason that some difference in our assays are not so profound. The expression of p62 can be regulated by various molecules with different mechanisms. Through the LC3-interacting region (LIR) domain, autophagy can bind p62 to LC3 on the membranes of autophagosomes and constantly degrade p62 via nonselective autophagy, thus playing a key role in the regulation of p62 levels [24]. Autophagy has routinely been suggested to potentiate the response to conventional therapies and inhibit tumour progression in gliomas [25]. 1L-6-hydroxymethylchiro-inositol 2(R)-2-O-methyl-3-O-octadecylcarbonate, an Akt inhibitor, reduces cell viability and radiosensitizes U87 glioma cells by inducing autophagy [26]. Imipramine can induce autophagy-associated cell death and reduce the incidence of gliomas in gliomagenesis models [27]. Gartanincan significantly induce autophagy and exhibit an anti-proliferation effect on T98G cells by inhibiting the PI3 K/Akt/mTOR signalling pathway [28]. Furthermore, NF-κB, Nrf2and AP1 can induce SQSTM1 gene transcription, forming a positive feedback for p62 transcriptional regulation. As a result, inflammation and oxidative stress can induce p62 through NF-κB and Nfr2 to promote cell detoxification and selective autophagy, leading to the prevention of cell death and tumorigenesis [29]. Guanylate-binding protein3 (GBP3) promotes cell growth through activating the p62-ERK1/2 signalling pathway in glioma [30]. Furthermore, we found that there is no difference in the expression of p62 between IDH wild type group and IDH mutated group in our experiment, indicating that p62 function may be independent of IDH status. However, this still needs to be verified in future work. Additionally, p62 can be regulated by other molecules, such as microRNAs.

MiR-124-3p has been confirmed to be downregulated in glioma tissues, and loss of miR-124-3p is associated with high malignance and poor prognosis in patients with glioma [31, 32]. Furthermore, the relative levels of miR-124-3p in serum exosomes could serve as a complementary diagnostic biomarker, providing a minimally invasive and innovative tool to diagnose gliomas at their onset and predict metastases and glioma grading before surgery [33]. Because a single miRNA may target hundreds of mRNA targets, miR-124-3p exerts anti-tumour functions by suppressing a variety of target genes. MiR-124-3p inhibits glioma cell proliferation by blocking the expression of STAT3 [34], iASPP [35], Smad4 [36], and Nur77 [37]. MiR-124-3p restoration represses the migration and/or invasion of glioma cells by targeting Capn4 [38], IQGAP1 [39], iASPP [35], and PIM1 [13]. By directly targeting N-Ras and R-Ras, miR-124-3p impairs angiogenesis through inhibiting VEGF transcription activation [40]. In this research, we demonstrate for the first time that miR-124-3p can directly regulate p62 in glioma. However, our experiment also has some flaws. The relatively low efficiency to inhibit miR-124-3p by anti-miR-124-3p may be the main reason why anti-miR-124-3p does not increase p62 levels considerably. In our future experiments, we will try to choose more efficient transfection tools to solve this problem.

## Conclusions

In sum, p62 can be targeted by miR-124-3p and acts as a tumour promotor in glioma by regulating autophagy, proliferation, migration, reactive oxygen species, TMZ resistance, glycolysis and NF-κB signalling pathway in glioma cells, suggesting the potential value of p62 as a novel therapeutic target for glioma.

## Supplementary information

**Additional file 1: Table S1.** Oligonucleotide sequences of siRNAs.

**Additional file 2: Table S2.** qRT-PCR primers for amplification of IDH, miR-124-3p, p62, CCL2, IL-6, TGFβ1 and CSF3.

**Additional file 3: Figure S1.** Relationship between IDH mutation and the expression of p62. (A) Statistical quantitation of the p62 expression in IDH wildtype tumours vs IDH mutant tumours. (B) Statistical quantitation of the p62 expression in IDH wildtype tumours vs IDH mutant tumours with different malignance. ns indicates not significant.

**Additional file 4: Figure S2.** Role of p62 overexpression in cell autophagy and NF-κB signalling pathway. (A) Relative p62 protein levels in U87 and U251 cells after transfection with nc-vector or p62-vector and treatment with CQ. (B) Statistical quantitation of the role of p62 overexpression in cell autophagy detected with western blot. (C) Left: Nuclear NF-κB protein levels in U87 and U251 cells after transfection with nc-vector or p62-vector detected with western blot. Right: Statistical analysis. The relative protein expression of NF-κB in nc-vector transfected U87 or U251 cells were arbitrarily set as 1. The results are presented as the mean ± SD of three independent experiments. (D to G) Relative mRNA levels of CCL2, IL-6, TGFβ1 and CSF3 in p62-overexpressed cells. *P < 0.05, **P < 0.01, ***P < 0.001, ns indicates not significant.

**Additional file 5: Figure S3.** Role of p62 knockdown in cell autophagy and NF-κB signalling pathway. (A) Relative p62 protein levels in U87 and U251 cells after transfection with nc-siRNA or p62-siRNAs and treatment with CQ. (B) Statistical quantitation of the role of p62 knockdown in cell autophagy detected with western blot. (C) Nuclear NF-κB protein levels in p62 KD cells detected with western blot. (D to G) Relative mRNA levels of CCL2, IL-6, TGFβ1 and CSF3 in p62 KD cells. **P < 0.01, ***P < 0.001, ns indicates not significant.

## Data Availability

The datasets analysed during the current available from the corresponding authors on reasonable request.

## Abbreviations

CNS: central nervous system
TMZ: temozolomide
ROS: reactive oxygen species
3′-UTR: 3′-untranslated region
qRT-PCR: quantitative real-time PCR
IDH: isocitrate dehydrogenase
CCL2: C–C motif chemokine ligand 2
TGFβ1: transforming growth factor beta 1
CSF3: colony stimulating factor 3
IL-6: interleukin 6
NF-κB: nuclear factor κB
CCK-8: Cell-Counting Kit 8
ECAR: extracellular acidification rate
CQ: chloroquine
LIR: LC3-interacting region
GBM: glioblastoma multiform
Keap1: Kelch-like ECH-associated protein 1
Nrf2: nuclear factor E2 related factor 2
