# Supplementary material for: p62 acts as an oncogene and is targeted by miR-124-3p in glioma
Source: Cancer Cell Int. 2019 Nov 6;19:280. doi: 10.1186/s12935-019-1004-x (PMC6836386; doi:10.1186/s12935-019-1004-x)
Supplement: Supplementary file 1 — Additional file 1: Table S1. Oligonucleotide sequences of siRNAs. [file 12935_2019_1004_MOESM1_ESM.doc]

Additional files 1. Table S1. Oligonucleotide sequences of siRNAs

| No. | Name | Sequence |
| --- | --- | --- |
| 1 | p62 siRNA 1# | F 5’-GGAGCACGGAGGGAAAAGA-3’ |
|  |  | R 5’-UCUUUUCCCUCCGUGCUCC-3’ |
| 2 | p62 siRNA 2# | F 5’-GGCUGAAGGAAGCUGCCUU-3’ |
|  |  | R 5’-AAGGCAGCUUCCUUCAGCC-3’ |
| 3 | p62 siRNA 3# | F 5’-CACUUCGGGUGGCCAGGAU-3’ |
|  |  | R 5’-AUCCUGGCCACCCGAAGUG-3’ |
| 4 | nc-siRNA | F 5’-GUACCGCACGUCAUUCGUAUC-3’ |
|  |  | R 5’-UACGAAUGACGUGCGGUACGU-3’ |
