# Supplementary material for: p62 acts as an oncogene and is targeted by miR-124-3p in glioma
Source: Cancer Cell Int. 2019 Nov 6;19:280. doi: 10.1186/s12935-019-1004-x (PMC6836386; doi:10.1186/s12935-019-1004-x)
Supplement: Supplementary file 2 — Additional file 2: Table S2. qRT-PCR primers for amplification of IDH, miR-124-3p, p62, CCL2, IL-6, TGFβ1 and CSF3. [file 12935_2019_1004_MOESM2_ESM.doc]

Additional files 2. Table S1. PCR primers for amplification of IDH, miR-124-3p, p62, CCL2, IL-6, TGFβ1 and CSF3.

| No. | Name | Sequence |
| --- | --- | --- |
| 1 | miR-124-3p | Cat.#, 4427012 |
| 2 | U6 | Cat.#, 4427975 |
| 3 | IDH1 | F 5’- CGGTCTTCAGAGAAGCCATT-3’ |
|  |  | R 5’- CACATTATTGCCAACATGAC -3’ |
| 4 | IDH2 | F 5’- AGCCCATCATCTGCAAAAAC-3’ |
|  |  | R 5’- CTAGGCGAGGAGCTCCAGT -3’ |
| 5 | p62 | F 5’-GATGAGGAAGATCGCCTTGG-3’ |
|  |  | R 5’-TCTGGCATCTGTAGGGACTG-3’ |
| 6 | CCL2 | F 5’- CAATCAATGCCCCAGTCACC -3’ |
|  |  | R 5’- CCTGAACCCACTTCTGCTTG -3’ |
| 7 | IL-6 | F 5’- CTGCAGCCACTGGTTCTGT -3’ |
|  |  | R 5’- CCAGAGCTGTGCAGATGAGT -3’ |
| 8 | TGFβ1 | F 5’- GGATAACACACTGCAAGTGG -3’ |
|  |  | R 5’- GAGCTGAAGCAATAGTTGGTG -3’ |
| 9 | CSF3 | F 5’- GCTTCCTGCTCAAGTGCTTA -3’ |
|  |  | R 5’- TTCCCAGTTCTTCCATCTGC -3’ |
| 10 | β-actin | F 5’-CCCTGGCACCCAGCAC-3’ |
|  |  | R 5’-GCCGATCCACACGGAGTAC-3’ |
