# Supplementary material for: p62 acts as an oncogene and is targeted by miR-124-3p in glioma
Source: Cancer Cell Int. 2019 Nov 6;19:280. doi: 10.1186/s12935-019-1004-x (PMC6836386; doi:10.1186/s12935-019-1004-x)
Supplement: Supplementary file 3 — Additional file 3: Figure S1. Relationship between IDH mutation and the expression of p62. (A) Statistical quantitation of the p62 expression in IDH wildtype tumours vs IDH mutant tumours. (B) Statistical quantitation of the p62 expression in IDH wildtype tumours vs IDH mutant tumours with different malignance. ns indicates not significant. [file 12935_2019_1004_MOESM3_ESM.doc]

Additional file 3. Figure S1. Relationship between IDH mutation and the expression of p62


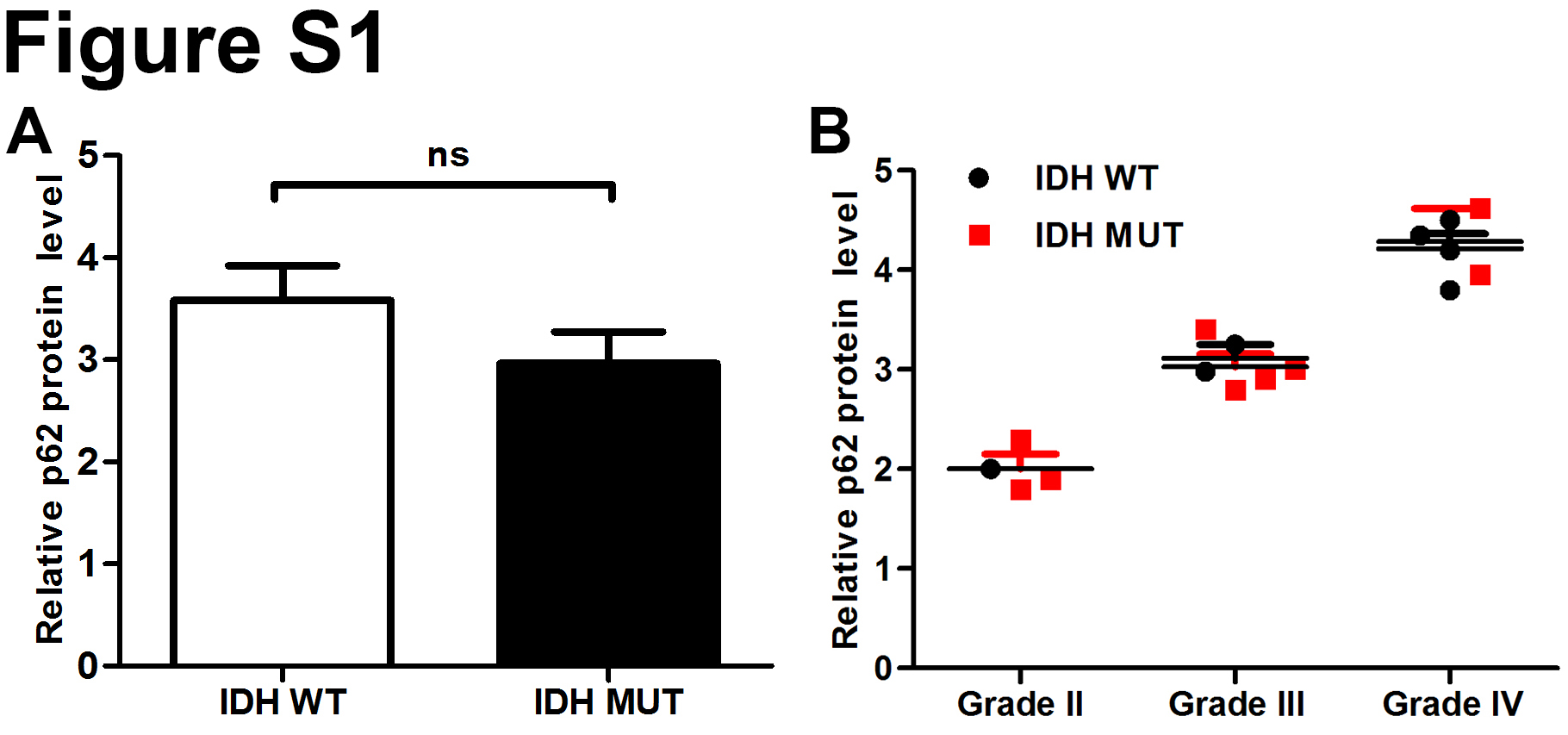


**Figure S1. Relationship between IDH mutation and the expression of p62.** (A) Statistical quantitation of the p62 expression in IDH wildtype tumours vs IDH mutant tumours. (B) Statistical quantitation of the p62 expression in IDH wildtype tumours vs IDH mutant tumours with different malignance. ns indicates not significant.
