# Supplementary material for: p62 acts as an oncogene and is targeted by miR-124-3p in glioma
Source: Cancer Cell Int. 2019 Nov 6;19:280. doi: 10.1186/s12935-019-1004-x (PMC6836386; doi:10.1186/s12935-019-1004-x)
Supplement: Supplementary file 4 — Additional file 4: Figure S2. Role of p62 overexpression in cell autophagy and NF-κB signalling pathway. (A) Relative p62 protein levels in U87 and U251 cells after transfection with nc-vector or p62-vector and treatment with CQ. (B) Statistical quantitation of the role of p62 overexpression in cell autophagy detected with western blot. (C) Left: Nuclear NF-κB protein levels in U87 and U251 cells after transfection with nc-vector or p62-vector detected with western blot. Right: Statistical analysis. The relative protein expression of NF-κB in nc-vector transfected U87 or U251 cells were arbitrarily set as 1. The results are presented as the mean ± SD of three independent experiments. (D to G) Relative mRNA levels of CCL2, IL-6, TGFβ1 and CSF3 in p62-overexpressed cells. *P < 0.05, **P < 0.01, ***P < 0.001, ns indicates not significant. [file 12935_2019_1004_MOESM4_ESM.doc]

Additional file 4. Figure S2. Role of p62 overexpression in cell autophagy and NF-κB signalling pathway


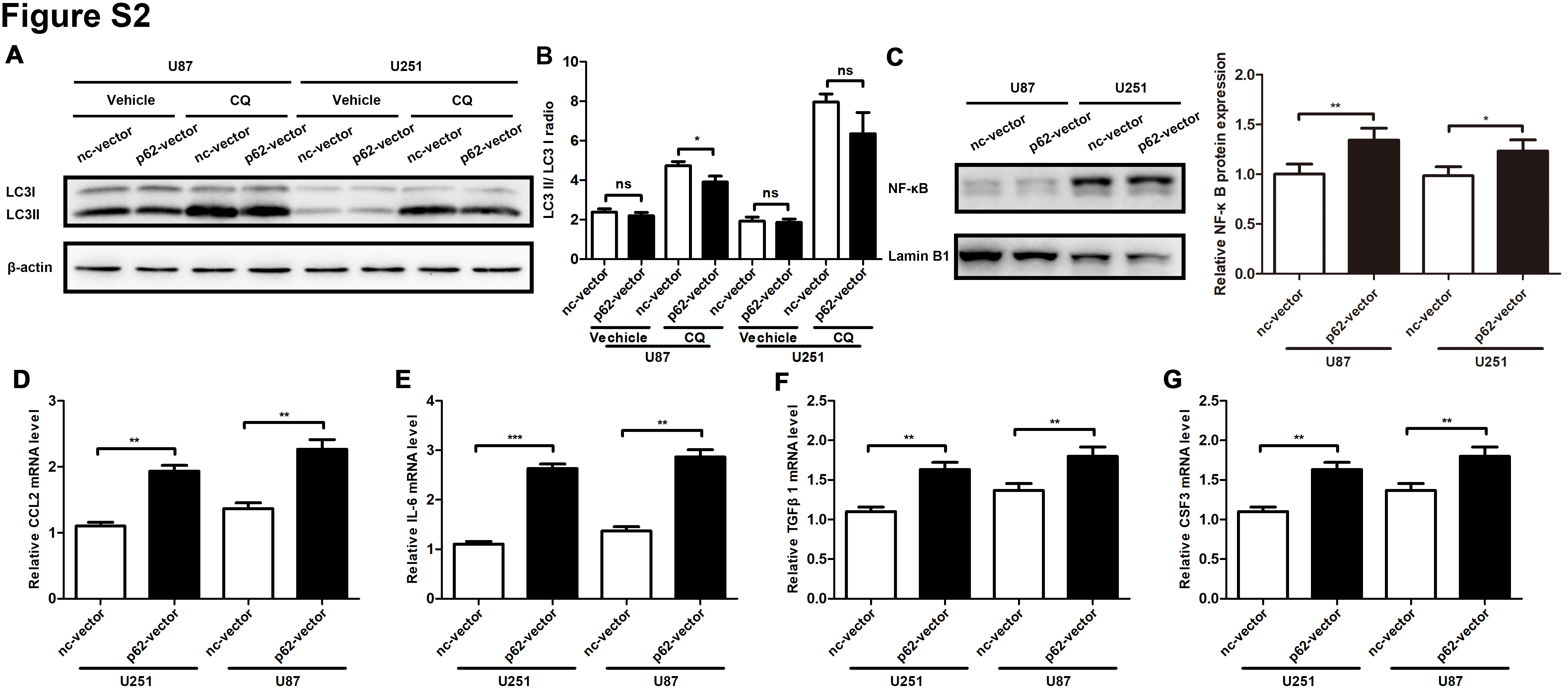


**Figure S2. Role of p62 overexpression in cell autophagy and NF-κB signalling pathway.** (A) Relative p62 protein levels in U87 and U251 cells after transfection with nc-vector or p62-vector and treatment with CQ. (B) Statistical quantitation of the role of p62 overexpression in cell autophagy detected with western blot. (C) Left: Nuclear NF-κB protein levels in U87 and U251 cells after transfection with nc-vector or p62-vector detected with western blot. Right: Statistical analysis. The relative protein expression of NF-κB in nc-vector transfected U87 or U251 cells were arbitrarily set as 1. The results are presented as the mean ± SD of three independent experiments. (D to G) Relative mRNA levels of CCL2, IL-6, TGFβ1 and CSF3 in p62-overexpressed cells. ^*^*P*< 0.05, ^**^*P*< 0.01, ^***^*P*< 0.001, ns indicates not significant.
