# Supplementary material for: p62 acts as an oncogene and is targeted by miR-124-3p in glioma
Source: Cancer Cell Int. 2019 Nov 6;19:280. doi: 10.1186/s12935-019-1004-x (PMC6836386; doi:10.1186/s12935-019-1004-x)
Supplement: Supplementary file 5 — Additional file 5: Figure S3. Role of p62 knockdown in cell autophagy and NF-κB signalling pathway. (A) Relative p62 protein levels in U87 and U251 cells after transfection with nc-siRNA or p62-siRNAs and treatment with CQ. (B) Statistical quantitation of the role of p62 knockdown in cell autophagy detected with western blot. (C) Nuclear NF-κB protein levels in p62 KD cells detected with western blot. (D to G) Relative mRNA levels of CCL2, IL-6, TGFβ1 and CSF3 in p62 KD cells. **P < 0.01, ***P < 0.001, ns indicates not significant. [file 12935_2019_1004_MOESM5_ESM.doc]

Additional file 5. Figure S3. Role of p62 knockdown in cell autophagy and NF-κB signalling pathway


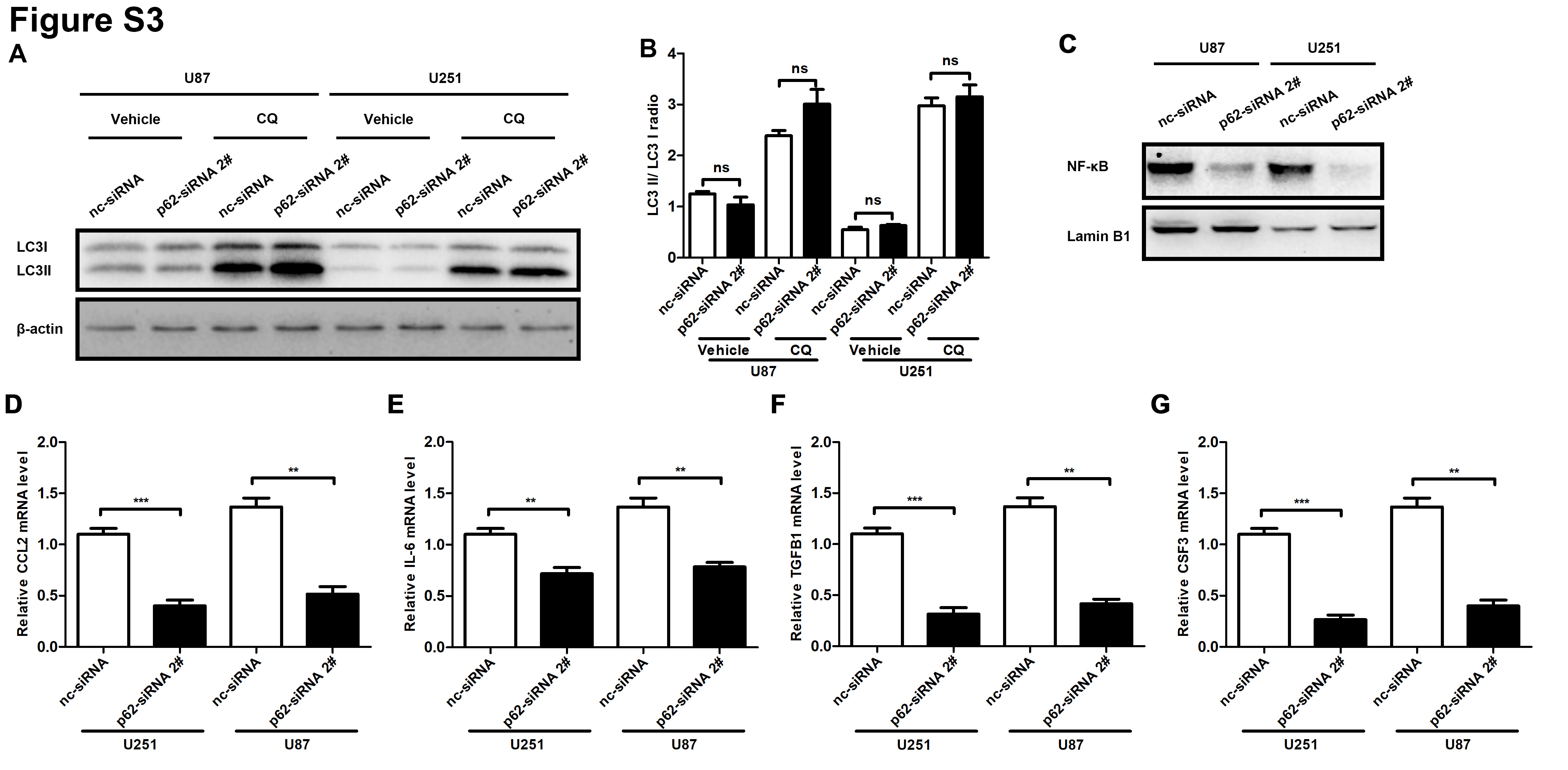


**Figure S3. Role of p62 knockdown in cell autophagy and NF-κB signalling pathway.** (A) Relative p62 protein levels in U87 and U251 cells after transfection with nc-siRNA or p62-siRNAs and treatment with CQ. (B) Statistical quantitation of the role of p62 knockdown in cell autophagy detected with western blot. (C) Nuclear NF-κB protein levels in p62 KD cells detected with western blot. (D to G) Relative mRNA levels of CCL2, IL-6, TGFβ1 and CSF3 in p62 KD cells. ^**^*P*< 0.01, ^***^*P*< 0.001, ns indicates not significant.
